# Acinar injury and early cytokine response in human acute biliary pancreatitis

**DOI:** 10.1038/s41598-017-15479-2

**Published:** 2017-11-10

**Authors:** Aparna Jakkampudi, Ramaiah Jangala, Ratnakar Reddy, Sasikala Mitnala, G. Venkat Rao, Rebala Pradeep, D. Nageshwar Reddy, Rupjyoti Talukdar

**Affiliations:** 1 Wellcome DBT Labs., Division of Basic Sciences, Asian Healthcare Foundation, New Delhi, India; 2 Department of Surgical Gastroenterology, Asian Institute of Gastroenterology, Hyderabad, India; 3 Department of Medical Gastroenterology, Asian Institute of Gastroenterology, Hyderabad, India

## Abstract

Clinical acute pancreatitis (AP) is marked by an early phase of systemic inflammatory response syndrome (SIRS) with multiorgan dysfunction (MODS), and a late phase characterized by sepsis with MODS. However, the mechanisms of acinar injury in human AP and the associated systemic inflammation are not clearly understood. This study, for the first time, evaluated the early interactions of bile acid induced human pancreatic acinar injury and the resulting cytokine response. We exposed freshly procured resected human pancreata to taurolithocolic acid (TLCS) and evaluated for acinar injury, cytokine release and interaction with peripheral blood mononuclear cells (PBMCs). We observed autophagy in acinar cells in response to TLCS exposure. There was also time-dependent release of IL-6, IL-8 and TNF-α from the injured acini that resulted in activation of PBMCs. We also observed that cytokines secreted by activated PBMCs resulted in acinar cell apoptosis and further cytokine release from them. Our data suggests that the earliest immune response in human AP originates within the acinar cell itself, which subsequently activates circulating PBMCs leading to SIRS. These findings need further detailed evaluation so that specific therapeutic targets to curb SIRS and resulting early adverse outcomes could be identified and tested.

## Introduction

Clinical acute pancreatitis (AP) is a burgeoning challenge to clinicians and scientists. The most common risk factors for AP are gallstones and alcohol; and the incidence of this potentially life-threatening illness has been increasing globally^1^. The clinical course of AP is broadly divided into two phases^2^. The initial phase that lasts for the first 1–2 weeks is characterised by systemic inflammatory response syndrome (SIRS) with or without multiorgan dysfunction, and is associated with the first peak of mortality. Pancreatic and peripancreatic necrosis evolves during this phase. The second phase that is seen from the second week onwards is marked by infections (including infected pancreatic necrosis [IPN]) in susceptible patients, and sepsis associated multiorgan dysfunction. This results in the second peak of mortality. Currently there is no curative treatment for this illness, and patient management is largely restricted to supportive care. Even though several treatment modalities have been shown to be beneficial in experimental AP in rodents, these have shown variable results in patients with AP^3^. In contrast to experimental studies on AP in rodents, where the pathophysiology has been elucidated in depth, mechanistic aspects of clinical AP in humans have been scantly studied. Few studies have described calcium signaling in human pancreatic acinar cell physiology; and a role of ryanodine receptors and dysregulated intra-acinar calcium signaling in mediating injury of human pancreatic acinar cells^4–6^. However, the interaction between human pancreatic acini and systemic inflammation, which is a determinant of morbidity and mortality in clinical AP, has remained largely speculative.

In this study we investigated the early interaction of human pancreatic acinar injury with inflammatory cytokines in the pathogenesis of AP. We used gallstone AP as a model since taurolithocolic acid (TLCS), a bile acid known to cause experimental AP in rodents^7,8^, has also been shown to cause injury to human pancreatic acinar cells via involvement of calcineurin and NF-κB^9^.

## Results

We procured 1–3 cm^3^ sized healthy pancreatic tissue from the transection margin of the intact pancreas from 88 patients [Mean (SEM) age 51.4 (1.6) yrs; both genders] who underwent either Whipple’s pancreaticoduodenectomy or distal pancreatectomy (DP) with or without splenectomy over a period of approximately 4.5 years (September 2012–June 2017). Indications for Whipple’s surgery/DP were pancreatic and duodenal neuroendocrine tumours, serous cystadenoma, indeterminate common bile duct stricture, symptomatic ampullary adenoma, periampullary mass, and IPMNs. Viability of acinar cell clusters was confirmed by Trypan blue method and those with >90% viability were used for experiments. Pancreatic tissue fragments with excessive fat, and those which showed autolysis or high LDH release even at the beginning of the experiments were discarded (data not shown). Of the 88 pancreatic samples procured, 63 could be used for experiments and data obtained from 52 of them were finally used for analyses.

### Morphological and functional characteristics of acini

**Figure 1:**
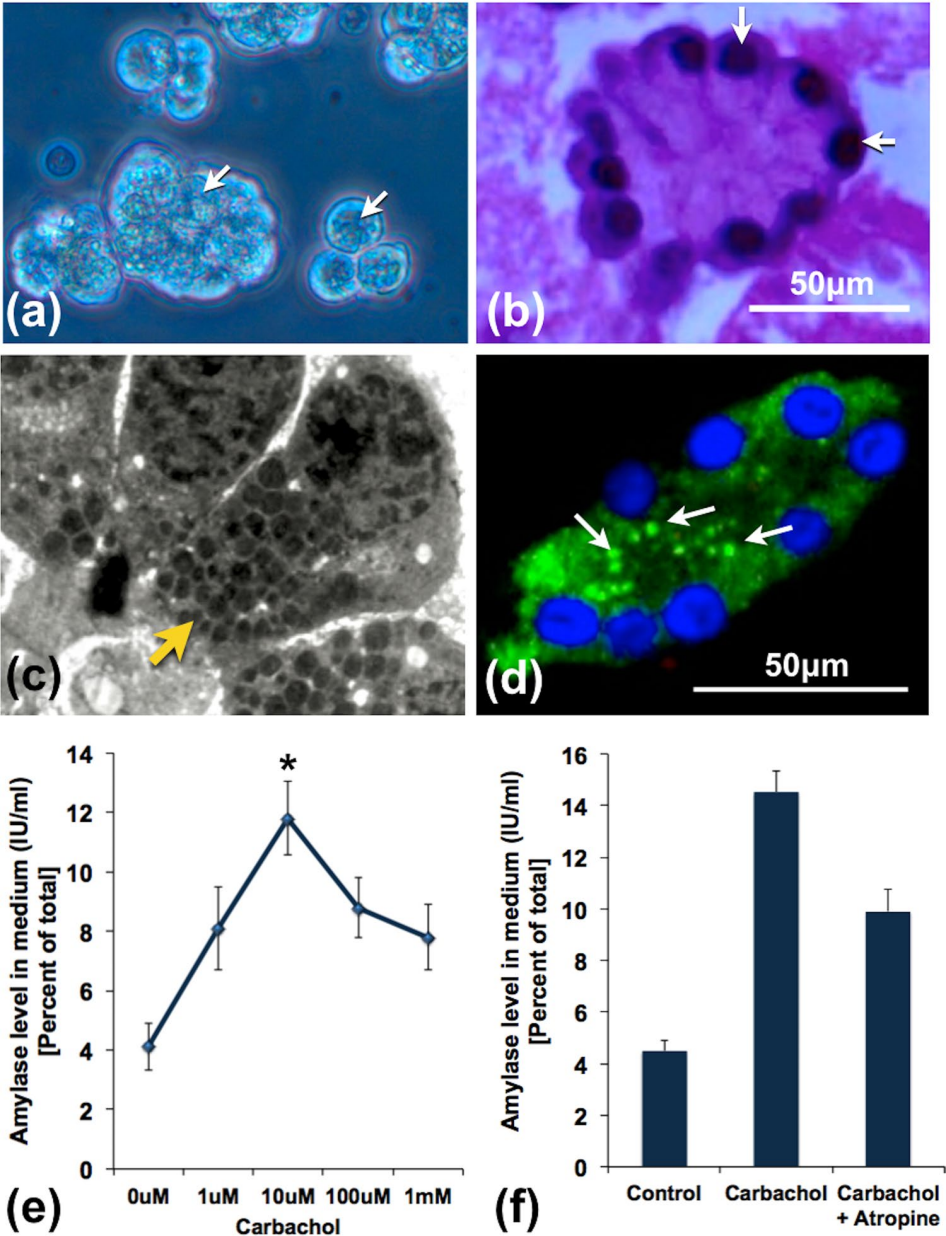
Structural and functional integrity of acini prior to induction experiments. (**a**) Inverted microscopic image of acinar clusters with apically located zymogen (white arrows). (**b**) H&E image showing an acinus with basally located nuclei (white arrows). (**c**) Transmission electron microscopic image showing part of an acinus with a polarized acinar cells showing basally located nuclei and apically located zymogen (yellow arrow). **(d**) IF image showing amylase stain within acinar cells distributed predominantly in the apical region (white arrows). (**e**) Line diagram representing dose dependent amylase secretion in response to stimulation with incremental doses of carbachol. Maximal amylase secretion was observed with 10 μM of carbachol, which was significantly higher compared to controls (*indicates p < 0.05). Thereafter, there was persistent reduction of amylase secretion with higher doses of carbachol. (**f**) Histograms showing the effect of atropine on amylase secretory response after stimulation of pancreatic acini with 10 μM carbachol. Pre-treatment of the acini with 10 μM atropine for 20 mins resulted in reduction in amylase secretion. The error bars indicate standard error of mean (SEM). The figures represent data from at least 3 independent experiments.

In order to assess the morphological and functional integrity of the procured acini, we subjected them to histological studies and exposed them to incremental doses of carbachol respectively. Figure 1a to d shows the healthy morphology of the procured acinar tissue fragments and clusters. As shown in Fig. 1c and d, there was apical distribution of zymogens, indicating that the acinar cells were polarized and were morphologically intact. When acinar fragments were exposed to increasing doses of carbachol for 30 mins, there was an incremental amylase secretory response up to a dose of 10 μM carbachol following which there was decline (Fig. 1e). Similar secretory responses were also reported in recent studies using human pancreatic acinar clusters and tissue slices^5,10^. We did not observe any incremental response after exposure for 15 mins (data not shown). The physiological response of the acinar cells to carbachol was further confirmed by inhibition of amylase secretion in the acini pre-treated by atropine when compared to responses to carbachol treatment alone.

### Human pancreatic acinar injury after exposure to bile acids

**Figure 2:**
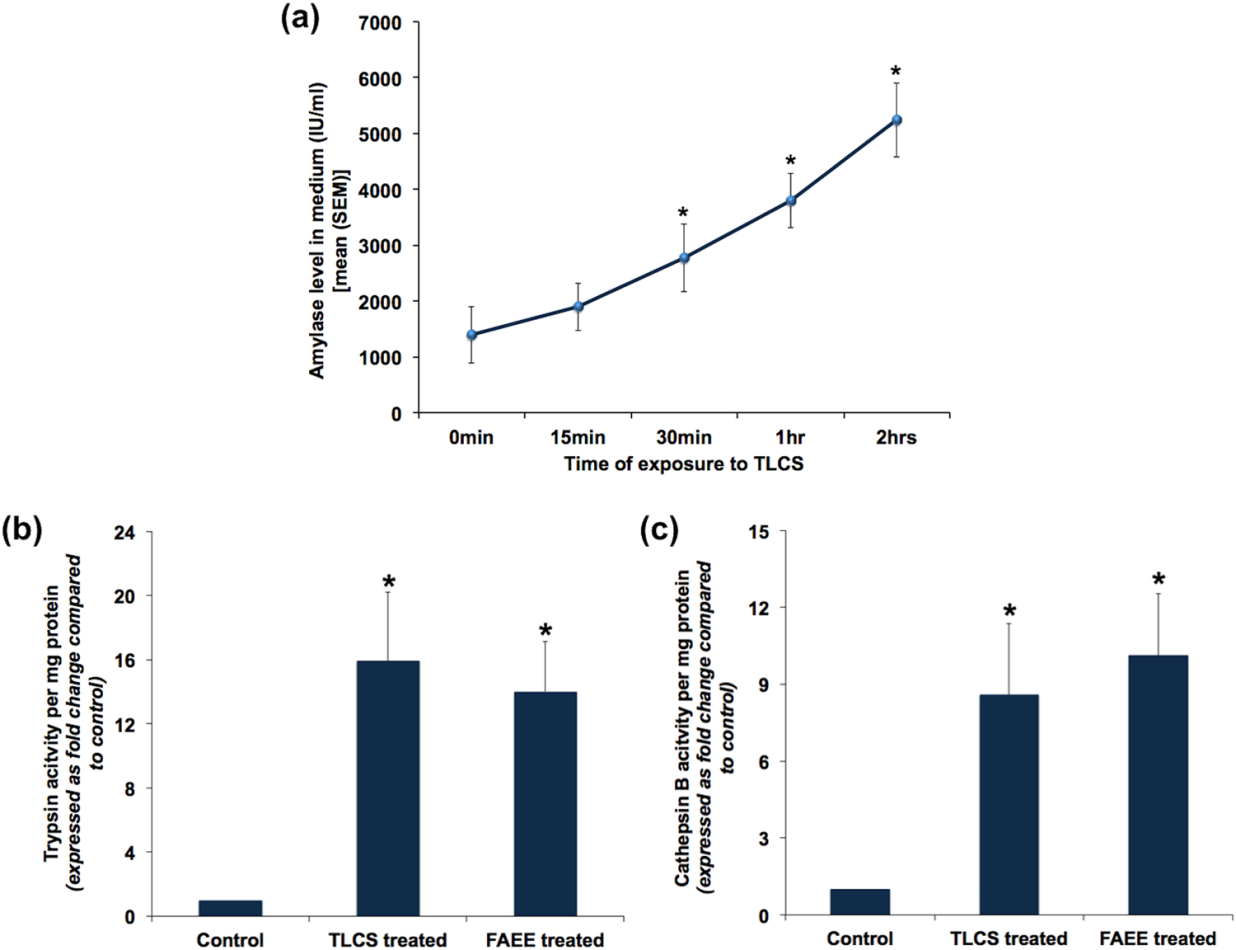
Human pancreatic acinar injury in response to exposure to TLCS and FAEE. (**a**) Time dependent increase in amylase secretion into medium after exposure of human pancreatic acinar tissue to 500 μM TLCS. Each point indicates the absolute value of amylase activity at each time-point. *Indicates p < 0.05 compared to 0 min. **(b)** and **(c)** Significant increase in trypsin and cathepsin B activities respectively at the end of 1 hr treatment of human pancreatic acinar tissue with 500 μM TLCS and 50 μM FAEE. Histograms indicate mean enzyme activities and error bars indicate standard error of mean. The figures represent data from at least 3 independent experiments. *Indicates p < 0.05 compared to control.

**Figure 3:**
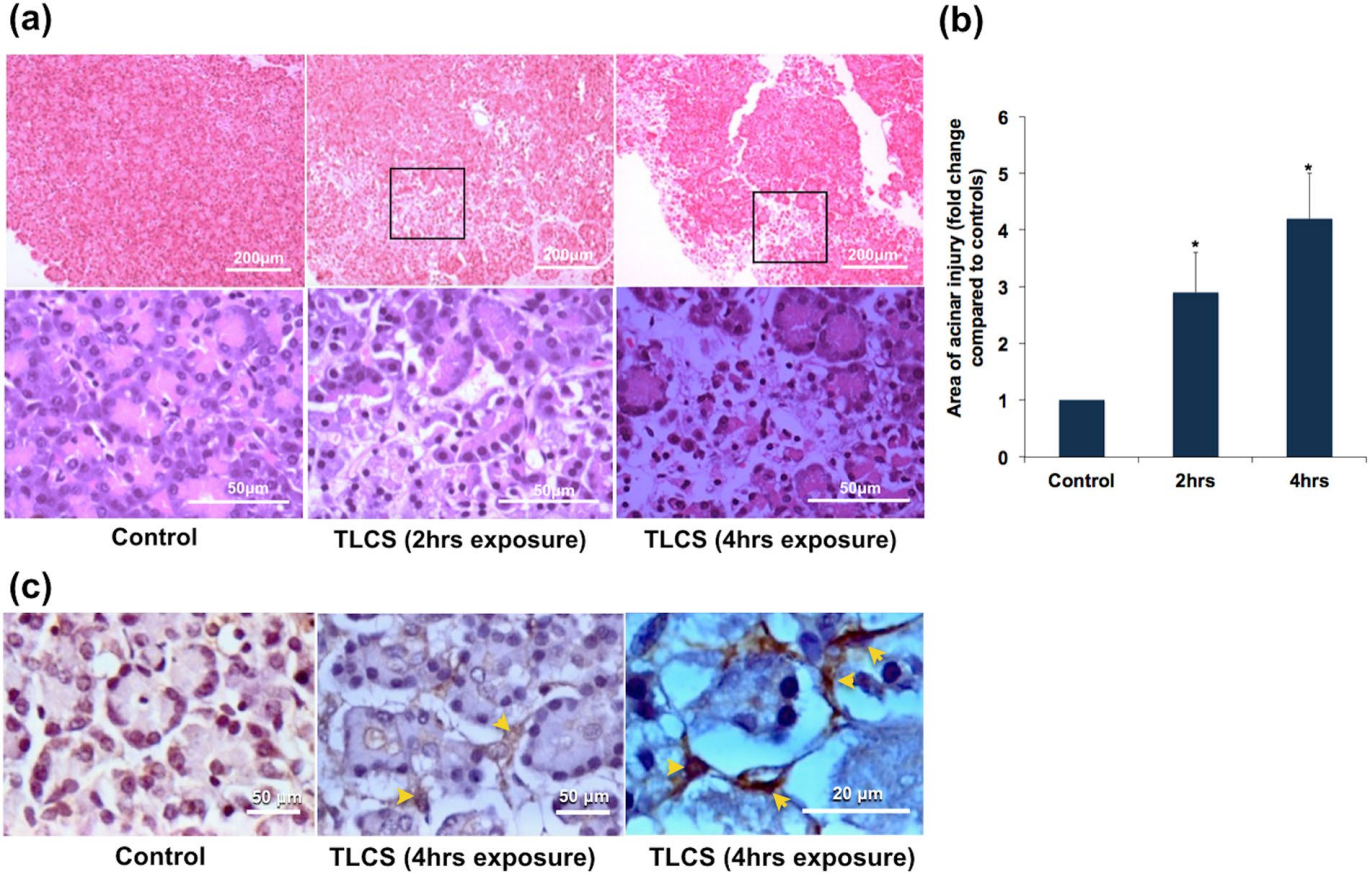
Histologic changes in human pancreatic tissue fragments in response to exposure to TLCS. Pancreatic tissue fragments were treated with TLCS and used for preparation of blocks for histology. These blocks were then sliced into thin sections of 140 μm and examined under microscope. (**a**) Representative H&E images showing injury to the pancreatic acini as evidenced by patchy areas of acinar loss after 2 and 4 hrs of exposure to 500 μM TLCS (upper panels)[scale bar 200 μ]. Magnified images of selected area (lower panels)[scale bar 50 μ] show loss of acinar cohesiveness and acinar cell necrosis. Since the experiments were performed *ex vivo* on pancreatic tissue fragments derived from operated samples, we did not expect signs of acute inflammation such as edema, vascular congestion, and inflammatory cell infiltration. **(b)** Histograms depicting the total area of pancreatic injury that progressively increased with increasing duration of exposure to TLCS. *Indicates p < 0.05 compared to control. (**c**) Representative IHC images of activated PSCs after expsure of human pancreatic acini with 500 μM TLCS for 4 hrs. **(a)** Controls, **(b)** and **(c)** Activated PSCs at different magnifications (scale bar 50 μ and 20 μ respectively). Activated PSCs are represented by α-SMA positive cells (brown staining) with a periacinar distribution (indicated by yellow arrows).

In order to confirm if bile acid causes injury to human pancreatic acini, we exposed pancreatic fragments to 500 μM of TLCS. In the standardization experiments, we conducted a dose response assay to identify the optimal dose of TLCS. Based on the results (Supplementary Fig. S1), we decided to use a dose of 500 μM for all subsequent experiments. This dose had also been used in earlier studies that evaluated the effect of TLCS in murine and human pancreatic acini and had shown to produce dose-specific injury^5,11^. We observed a time dependent increase in amylase secretion into the medium, which reached statistical significance after 15 mins of exposure (Fig. 2a). There was also significant increase in trypsin and cathepsin B activities after exposure to TLCS for 1 hr (Fig. 2b and c respectively), which suggest intrapancreatic trypsin activation. In order to confirm if our results with TLCS were not non-specific, we also treated acinar cells with FAEE and caerulein. FAEE and caerulein are noxious stimuli known to cause acute pancreatitis^4,12^. As shown in Fig. 2b and c, there was a similar increase in trypsin and cathepsin B activities respectively with FAEE, akin to that with TLCS. In the experiments with caerulein, we observed a statistically significant increase in trypsin and cathepsin B activity in two out of six experiments with acinar cell preparations. In the remaining four experiments, even though there was increased activity, it was not significantly high. Acinar injury was confirmed by H&E staining of TLCS exposed tissue that showed swollen and lightly stained cytoplasm, nuclear pyknosis, loss of membrane and loss of acinar cells in pancreatic tissue fragments. Compared to controls, the injury was nearly 3 folds when exposed to TLCS for 2 hrs while it increased to over 4 fold after exposure for 4 hrs (Fig. 3a and b). Since the experiments were conducted *ex vivo*, we did not expect to find features of acute inflammation such as edema, vascular congestion and inflammatory cellular infiltration.

We also observed pancreatic stellate cell (PSC) activation as marked by the presence of α-SMA positive cells with a peri-acinar distribution, which also marks the presence of pancreatic injury (Fig. 3c).

### Bile acids mediated human pancreatic injury is associated with intra-acinar vacuole formation

**Figure 4:**
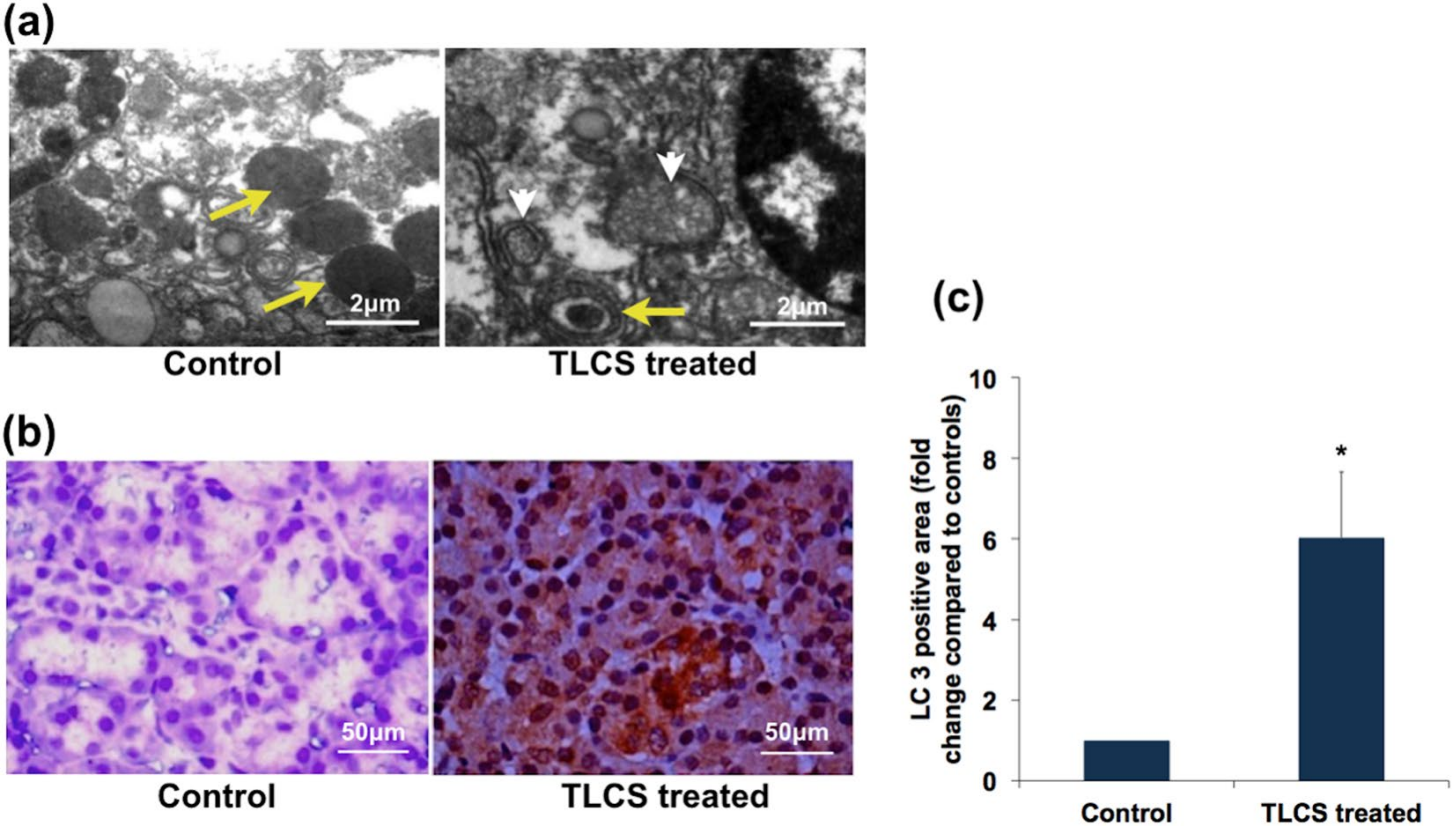
Autophagy in human pancreatic acini in response to exposure to TLCS. (**a**) Representative transmission electron microscopic (TEM) images depicting autophagy. The control panel shows normal acinar zymogens (yellow arrow), while the 1-hr TLCS treated acini shows smaller zymogen within a doubled membrane vesicle (yellow arrows), and degraded cellular material within double membrane vesicular structures (white arrow heads) indicating different stages of autophagolysosomes [scale bar 2 μ]. **(b)** Representative immunohistochemistry (IHC) images showing substantial cytosolic staining for LC3 within the acinar cells on treatment with 500 μM TLCS (as indicated by brown staining) compared to controls acinar tissue [scale bar 50 μ]. **(c)** Histogram depicting the quantitative representation of LC3 staining (fold change in LC3 positive area compared to control) in the acinar tissues as per methods described in the *materials and method* section. (*Indicates p = 0.005 compared to control; error bar indicates standard error of mean [SEM]).

Previous studies in experimental AP using rodent models had demonstrated intra-acinar vacuoles that were variably named co-localized organelles or autophagic vacuoles^13–15^. We conducted transmission electron microscopic (TEM) studies to evaluate if similar vacuole formation occurred in human biliary AP. On treatment with 500 μM TCLS, we observed doubled membrane vacuoles of varying size containing zymogen and partially degraded material/debris (Fig. 4a). These characteristics suggested that these were autophagic vacuoles. In order to confirm this, we further evaluated for the specific marker of autophagy, LC3, which was expressed 6-folds greater in the acinar cells from TLCS treated tissue fragments compared to controls (Fig. 4b and c).

### Cytokine secretion by pancreatic acinar cells in response to bile acid injury

**Figure 5:**
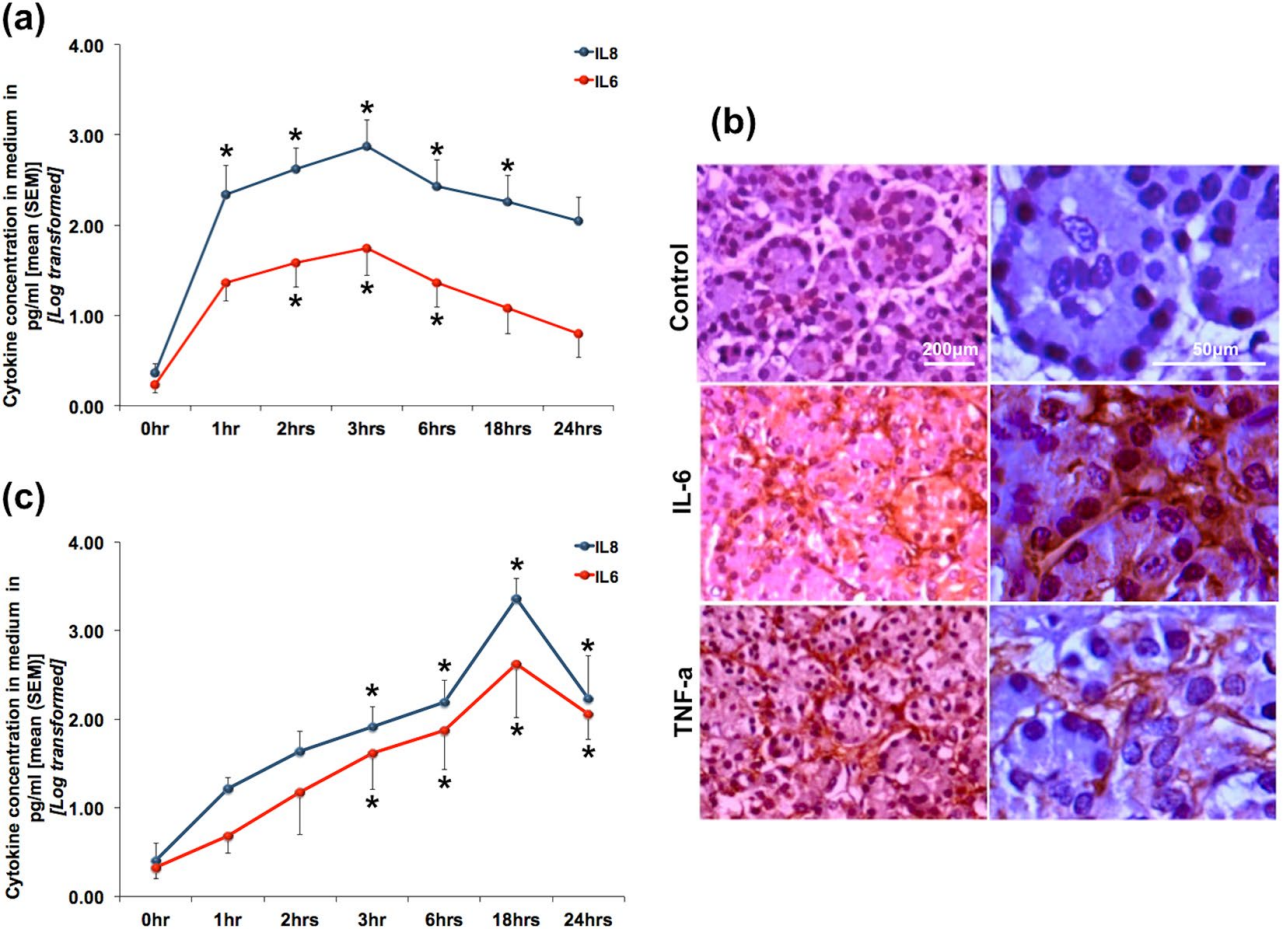
Cytokine release by human pancreatic acini in response to exposure to TLCS and FAEE. Line diagrams showing time dependent secretion of IL-6 and IL-8 into the medium after exposure to **(a)** 500 μM TLCS and **(c)** 50 μM FAEE. While peak level of cytokine in response to TLCS was reached after exposure for 3 hrs, that in response to FAEE was reached at 18 hrs, after which there was a gradual reduction. (*Indicates p < 0.05 compared to 0 hr). (**b**) Representative IHC images showing IL-6 within the pancreatic acini and TNF-α localized to the basolateral aspects of the acinar cells. Panels on the left represent lower magnification [scale bar 200 μ], while the panels on the right are higher magnifications [scale bar 50 μ] of the same.

Even though AP originates primarily within the pancreatic acini, clinical outcomes appears to be determined by the degree of systemic inflammatory response syndrome (SIRS) and organ dysfunction^16,17^. Since cytokines are responsible for mediating SIRS, we evaluated the relationship of pancreatic acinar injury and cytokine response. As shown in Fig. 5a, there was a time dependent increase in the secretion of IL-8 and IL-6 from the pancreatic tissue fragments into the medium that reached peak levels after 3 hours of exposure to TLCS. We further confirmed expression of IL-6 and TNF-α within the acini by IHC, where we observed IL-6 in an intra-acinar location and TNF-α, which localized to the basolateral region of the acinar cells (Fig. 5b). As shown in Fig. 5c, even with stimulation with FAEE there was a time dependent increase in IL-6 and IL-8 secretion into the medium, which reached peak at 18 hrs after induction. This observation ruled out the possibility of a non-specific effect of TLCS.

**Figure 6:**
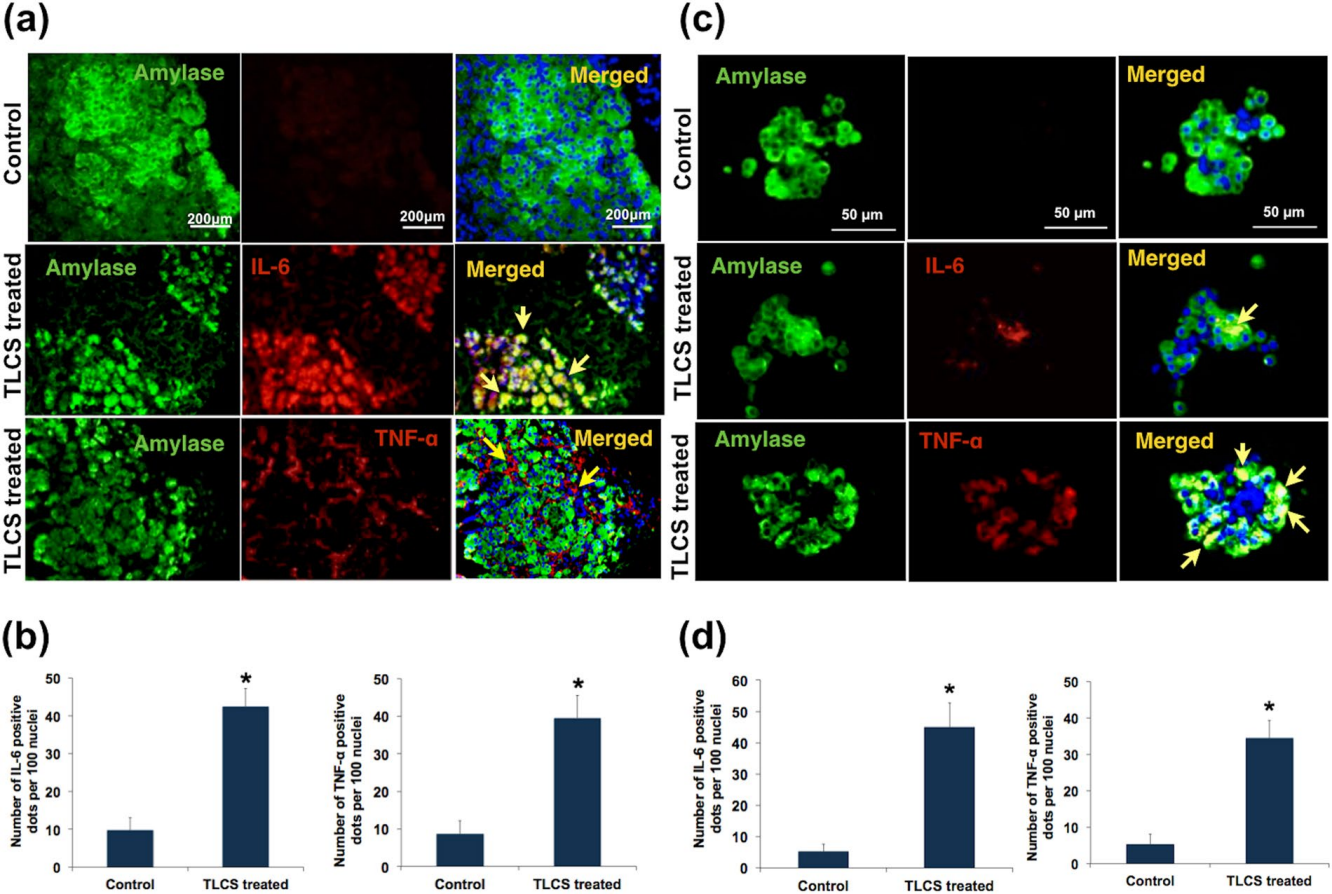
Confirmation of cytokine release by human pancreatic acini in response to exposure to TLCS. **(a,c**) Representative immunofluorescence (IF) images [scale bar 200 μ] indicating IL-6 and TNF-α after treatment with 500 μM TLCS for 3 hrs (**a**) from experiments using acinar tissue while **(c)** from experiments using isolated acini. Green fluorescence in the panels indicates amylase, while red fluorescence indicates IL-6 and TNF-α in the panels of second and third rows respectively. Yellow fluorescence in the merged images in the second row indicates co-localization of IL-6 with amylase implying the location of IL-6 within the acini. Linear red fluorescence around the basolateral surface of the acinar cells in the merged image of the third row indicates localization of TNF-α along the basolateral surface of acinar cells. **(b)** Histograms showing the quantitative representation of IL-6 and TNF-α positivity respectively, which was significantly higher than that of controls (p = 0.017 and 0.018 respectively) in acinar tissue. **(d)** Histograms showing the quantitative representation of IL-6 and TNF-α positivity respectively, which was significantly higher than that of controls (p = 0.004 and 0.003 respectively) in isolated acini. Histograms and error bars in **(b)** and **(d)** indicate mean standard error of mean (SEM) respectively.

In order to prove that the cytokines were secreted primarily by the acinar cells, and not by any other potentially resident immune cells in the tissue fragments, we conducted IF studies using dual staining for amylase and cytokines (IL-6 and TNF-α). As shown in Fig. 6a, IF using pancreatic tissue fragments showed co-localization of IL-6 with amylase indicating the intra-acinar location. TNF-α was found to be localized to the basolateral surface of the acini. We could replicate these results in isolated pancreatic acinar clusters, thereby confirming cytokine secretion by the pancreatic acinar cells in response to injury by bile acids (Fig. 6c). As shown in Supplementary Fig. S2, IL-6 and TNF-α were secreted by the TLCS treated acinar cells for up to 6 hrs post exposure.

### Effect of acinar injury on PBMCs

**Figure 7:**
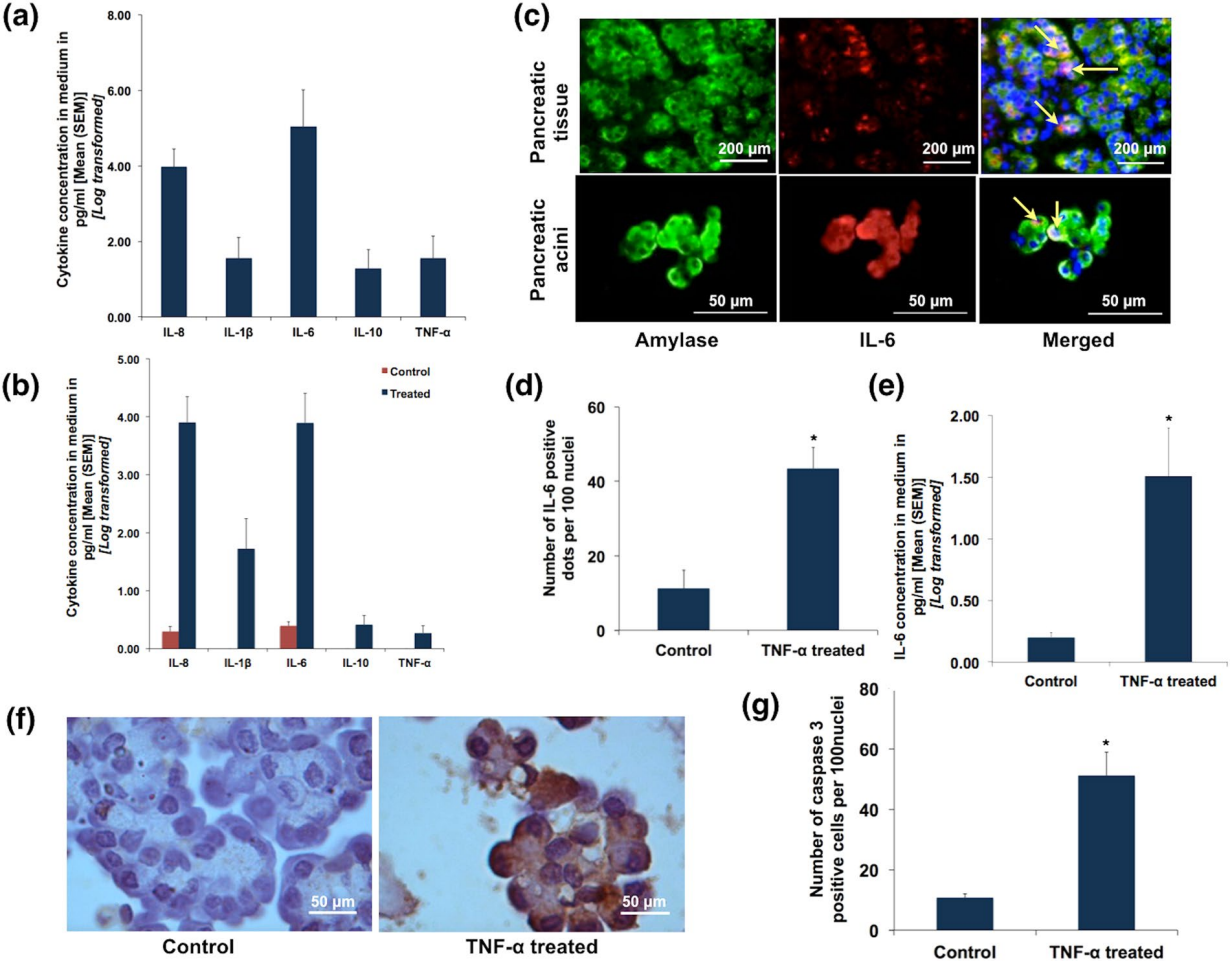
Interaction between acini and cytokine secretion. **(a)** Histograms showing quantification of cytokines secreted by PBMCs upon exposure to conditioned media from TLCS treated pancreatic acinar tissue. Pancreatic acinar tissues were treated with 500 μM TLCS for 3 hrs and the medium was collected and used to treat freshly processed PBMCs from the same individual. **(b)** Histograms showing quantification of cytokines secreted by acinar tissue upon exposure to conditioned media from LPS treated PBMCs. PBMCs were collected from individuals prior to resection and treated with LPS and incubated for 12 hrs. The medium was then used to treat acinar tissue from the same individual that was obtained after resection *(refer to methods section for experimental details)*. **(c)** Representative IF images depicting IL-6 secretion from pancreatic tissue (upper panel) and pancreatic acini (lower panel) on treatment with recombinant TNF-α (100 ng/ml). Green fluorescence in the panels indicates amylase, while red fluorescence indicates IL-6. Orange/yellow fluorescence (indicated by yellow arrows) in the merged images indicates co-localization of IL-6 with amylase implying the localization of IL-6 within the acini. **(d**) Histogram quantifying IL-6 positivity in recombinant TNF-α treated acinar tissue. (**e**) Histogram quantifying the concentration of IL-6 secreted by acinar tissue into the medium upon treatment with recombinant TNF-α. **(f)** Representative IHC images depicting caspase 3 positive acinar cells after treatment with recombinant TNF-α [scale bar 50 μ]. **(g)** Quantitative data showing significantly higher caspase 3 positive acinar cells compared to controls after treatment with recombinant TNF-α (p = 0.02). Histograms and error bars in **(a)**, **(b**,**d**,**e)** and **(g)** indicate mean and standard error of mean (SEM) respectively.

We next evaluated the effect of acinar injury on PBMCs, for which we treated isolated PBMCs with conditioned media from TLCS treated acinar clusters. The PBMCs were collected from the patients before pancreatic resection. We observed robust secretion of IL-8 and IL-6, along with secretion of TNF-α and IL-10 (Fig. 7a).

### Effect of cytokines on pancreatic acini

We then evaluated the effect of cytokines on the pancreatic acini. For this, we initially treated PBMCs from the same individual with lipopolysaccharide (LPS) and confirmed the secretion of cytokines (data not shown). We used this conditioned medium to treat normal pancreatic fragments obtained after resection. This resulted in secretion of IL-8, IL-1β and IL-6 (Fig. 7b). In order to confirm acinar cell injury and further cytokine release from them in response to cytokine containing media, we treated healthy pancreatic fragments and acinar clusters with recombinant TNF-α. We observed significant expression of IL-6 within the acinar cells in both preparations (acinar tissue fragment and isolated acinar clusters), thereby indicating that TNF-α could result in further acinar injury (Fig. 7c and d). This was further supported by our observation that there was significantly higher IL-6 secretion into the media where healthy acini were treated with recombinant TNF-α (Fig. 7e). As shown in Fig. 7f and g, there was also apoptosis as depicted by caspase 3 staining in recombinant TNF-α treated healthy acinar cells. This reconfirmed that cytokines caused further injury to the pancreatic acinar cells.

### Cytokine secretion in the early stage of acute biliary pancreatitis in patients

**Figure 8:**
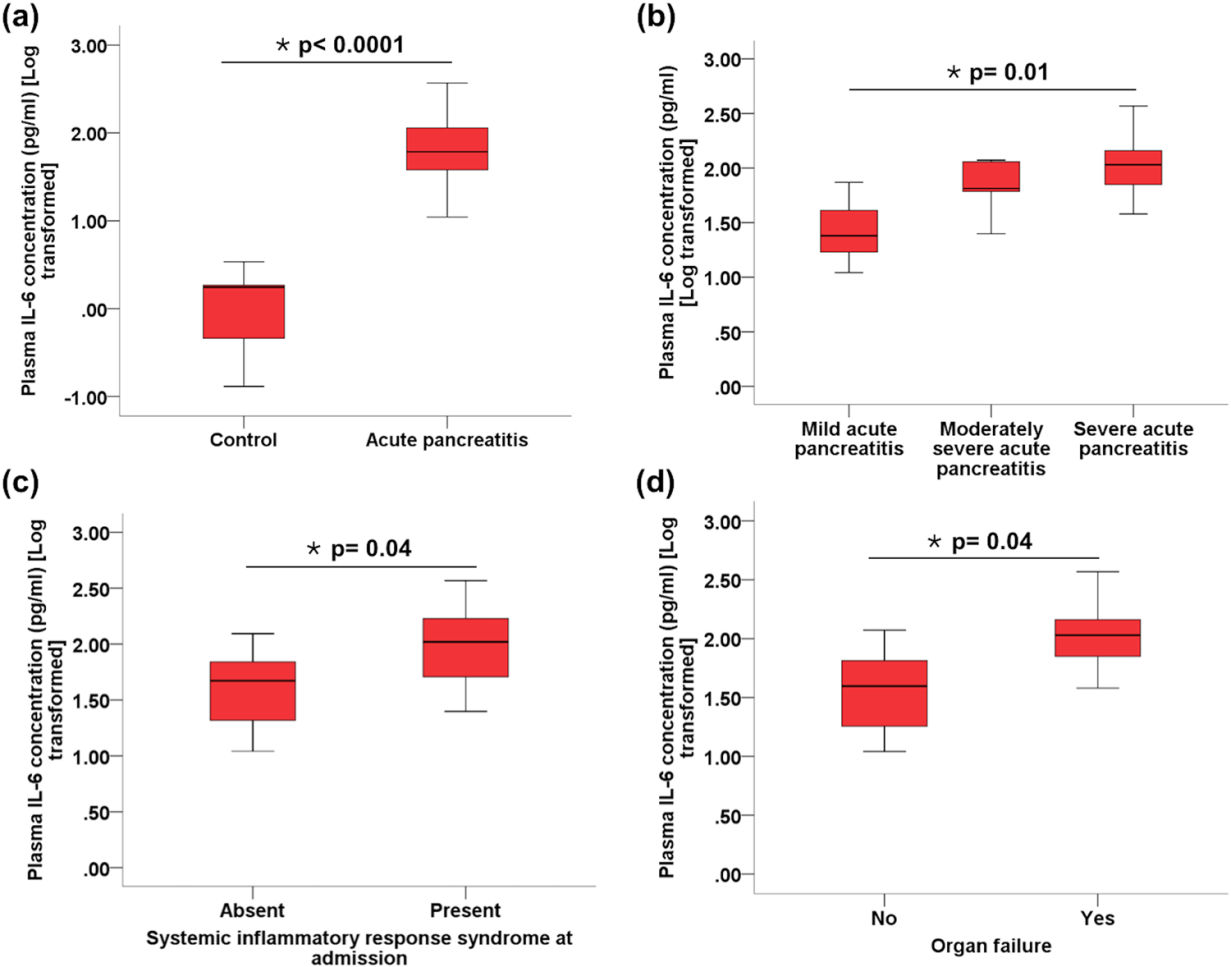
Box and whisker plots showing IL-6 concentration between. (**a**) healthy controls and patients with AP, **(b**) different grades of severity of AP, **(c**) patients with AP who had SIRS and those who did not, and **(d**) patients with AP who had organ failure and those who did not.

Table 1 shows the patient characteristics. All patients were admitted within 72 hrs of disease onset. As per the Revised Atlanta Classification, 17 (37.8%) patients had MAP, 13 (28.9%) had MSAP while 15 (33.3%) developed SAP. Six out of 15 patients with SAP developed organ failure within the first week of disease (four of whom had organ failure at the time of admission). These patients were defined as having early severe AP (ESAP), as proposed earlier; and two (33.3%) of these patients died in hospital. Twelve (48%) patients developed infected pancreatic necrosis and required percutaneous drainage with or without subsequent endoscopic (EUS guided) drainage. As shown in Fig. 8a, IL-6 concentration at the time of admission was significantly elevated in patients with biliary AP compared to healthy controls. Furthermore, IL-6 concentration in circulation progressively increased from mild to moderately severe to severe AP (Fig. 8b). It was also significantly elevated in patients who had systemic inflammatory response syndrome and organ failure at the time of presentation (Fig. 8c and d). Supplementary Fig. S3 shows representative FACS images of the cytokine panel in patients MAP, MSAP and SAP. Besides IL-6, there was also elevation of IL-8 in patients with MAP and MSAP. Furthermore, there was also a significant elevation of TNF-α in the patients with ESAP who died.

**Table 1:** Patient characteristics.

Parameter		Value (N = 45)
Age in years (Mean ± SD)		40.5 ± 17.6
Male gender (n; %)		34 (75.6)
Systemic inflammatory response syndrome (SIRS) at admission (n; %)		17 (37.8)
Severity	Mild (n; %)	17 (37.8)
	Moderately severe (n; %)	13 (28.9)
	Severe (n; %)	15 (33.3)
Renal failure (n; %)		11 (24.4)
Circulatory failure (n; %)		2 (4.4)
Respiratory failure (n; %)		8 (17.8)
In-hospital mortality (n; %)		2 (4.4)

## Discussion

Clinical AP is characterised by an early phase of SIRS and organ dysfunction^2^. This could result in early severe (ESAP) and fulminant AP^18,19^, which are marked by high mortality. This is followed by a second phase characterised by infections (including IPN) that results in a second wave of death. Even though several clinical studies have reported associations of circulating cytokines with adverse outcomes of AP^17,20^, data on the mechanistic aspects of clinical AP and the related systemic inflammation are scant. *Ex vivo* studies using human pancreatic tissue and acinar preparations have demonstrated the role of dysregulated intra-acinar calcium signaling in pancreatic acinar injury^6,7^. However, to the best of our knowledge there are no experimental studies that have evaluated the immune response after induction of human pancreatic acinar injury. This formed the basis of our current studies where we evaluated the key early events and the interaction with cytokines following exposure of pancreatic acinar cells to noxious stimuli. We used TLCS as noxious stimuli since bile acid mediated injury has been documented earlier in human pancreatic acini^9^.

We observed acinar injury in response to TLCS as marked by trypsin and cathepsin B activation and confirmed by H&E staining. We conducted preliminary experiments to evaluate a dose-response effect with TLCS and could demonstrate trypsin activation at a specific dose of 500 μM, thereby negating the possibility of a non-specific response to TLCS. Previous studies also demonstrated noxious effect of TLCS at a specific dose of 500 μM^5,11^. We further ruled out non-specific action of TLCS by using another known noxious stimuli, FAEE, in a few experiments in which we observe acinar injury and cytokine release.

We also observed early formation of autophagic vacuoles or autophagosomes as shown by TEM studies and confirmed this by the presence of the marker of autophagy (LC3). Impaired autophagy was earlier reported in human alcoholic pancreatitis as evidenced by depletion of the lysosomal marker LAMP2^21^. Impaired autophagy was also shown earlier in caerulein AP in rodents by Mareninova *et al*. where trypsin activation was seen within the autophagic vacuoles^15^. It appears likely from our results that perturbation of intracellular trafficking of zymogen may occur in human pancreatic acini in response to bile acid exposure too.

We further observed time-dependent increase in pro-inflammatory cytokine secretion along with the formation of autophagic vacuoles. IL-6 and IL-8 secretion into the medium was maximum after 3 hrs of induction of pancreatic injury to pancreatic tissue fragments with TLCS. In order to identify the source of pro-inflammatory cytokines in the media, we subjected the pancreatic tissue fragments and acinar cells clusters to IHC and IF for IL-6 and TNF-α, which revealed the source of these cytokines to be the acinar cells. TNF-α was earlier demonstrated in pancreatic acini in resected pancreata from three patients with AP and recurrent AP (n = 3; alcoholic, ischaemic and idiopathic)^22^. In our study, we have experimentally proven that the acinar cells indeed produce pro-inflammatory cytokines on exposure to bile acid. Even though it may be argued that cytokine release from the TLCS exposed acini could have been due to diffusion from dead acinar cells, the very observation that there was a time dependent increase in release with a peak at 3 hrs suggests that the early release is secretory in nature in response to injury. This is further supported by the observation that the acini were not grossly damaged at the time point in which the cytokine staining was conducted (Fig. 5b).

**Figure 9:**
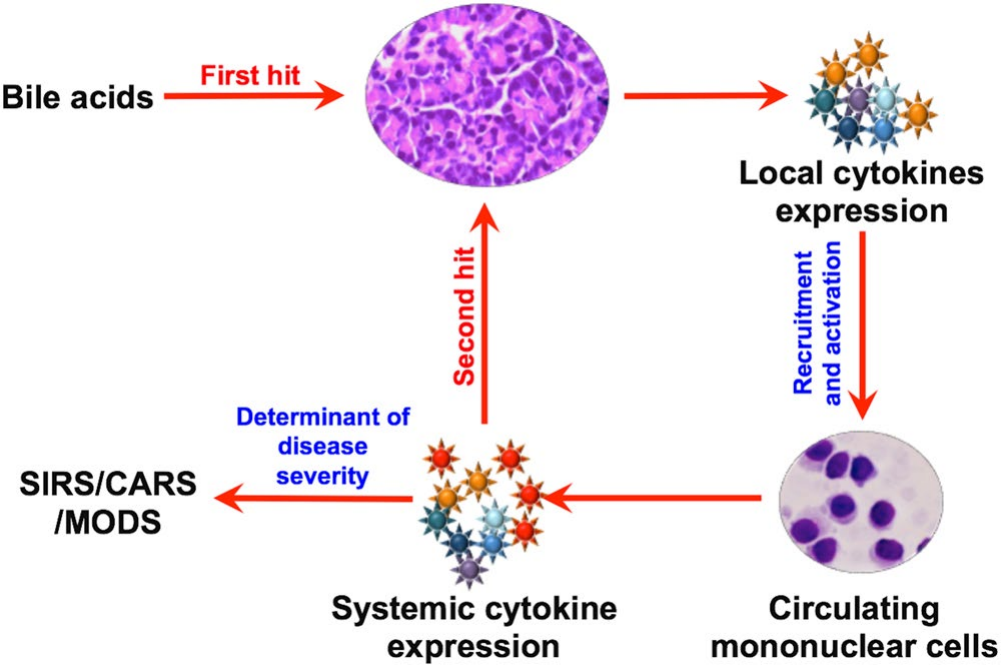
Schematic representation of the proposed sequence of early events in acute biliary pancreatitis in humans. Based on the results of our experiments, bile acid exposure to the acini acts as the first hit that results in acinar injury and intrapancreatic secretion of proinflammatory cytokines by the acinar cells. The released cytokines recruits and activates PBMCs within the pancreas. These activated PBMCs enter into the systemic circulation and further releases cytokines that can lead to a second hit to the pancreatic acinar cells and also cause early SIRS and MODS.

Since AP begins with intrapancreatic trypsin activation but the severity appears to be dependent on the systemic inflammation and organ dysfunction, we evaluated the interaction between TLCS exposed acini and PBMCs. On treatment of PBMCs with conditioned medium of TLCS treated pancreatic tissue fragments we observed robust pro-inflammatory cytokine response from the exposed PBMCs. The conditioned media, which contained cytokines, digestive enzymes and constituents of zymogen, mimicked the intrapancreatic inflammatory milieu to which the circulating PBMCs are recruited and exposed. We further exposed normal pancreatic acini to conditioned medium from LPS treated PBMC, where we observed cytokine secretion by the exposed acini. LPS is known to activate PBMCs that results in an inflammatory response^23^, and our observations suggest that cytokines could cause additional injury to the pancreatic acini. In order to confirm that this second hit to the acini is actually due to cytokines secreted by PBMCs, we treated normal pancreatic tissue fragment and acinar clusters with recombinant TNF-α. Besides secretion of IL-6 in response to recombinant TNF-α exposure, we also observed apoptosis in the acinar cells. TNF-α is known to cause apoptosis in acinar cells in experimental pancreatitis in rodents^24–26^. Our observations confirmed that TNF-α resulted in a second hit to the human pancreatic acinar cells subsequent to the first hit with TLCS. Figure 9 depicts the schematic representation of interaction of acinar injury and PBMCs.

In clinical practice, it is observed that generally 20–25% of patients with AP develop moderately severe to severe disease^27^; while one third of patients with severe disease develop ESAP^28^. Even though overall mortality is around 5%, it rises to 43% among patients who develop infected necrosis along with multiorgan failure^29^. Mortality among patients with early SAP (including fulminant AP) is very high (37.5–44%) and occurs even in the absence of infections and sepsis^18,19,28^. This is likely to result from the cytokine storm induced persistent multiorgan failure. In order to evaluate if our experimental finding extrapolates to patients with AP, we evaluated plasma cytokines in patients with biliary pancreatitis who were admitted within 72 hrs of onset of pain. IL-6 was significantly higher in patients who had SIRS and severe AP. Highest level of cytokines was observed in the two patients who died, and both had ESAP. Based on our results we believe that it is the magnitude of the second hit to the pancreas and the second wave of cytokine release from activated PBMCs that determines the severity of AP.

Over the past several decades, intra-pancreatic trypsin (protease) activation has been the central dogma in the pathogenesis of AP. Based on this premise, experimental studies had evaluated modalities to inhibit protease activation (eg. gabaxate mesilate, nafamostate) and pancreatic acinar secretion (somatostatin, octreotide). Even though these strategies have been effective in the experimental setting, results have been variable when tested in RCTs on patients with AP^3^. It is important to realize that in clinical AP, by the time patients present to the hospital the early event of protease activation is already past. Moreover, our recent studies using knock-out mice had shown that even though trypsin activation is important for acinar injury it is not always mandatory; and that trypsin may not be directly responsible for inflammation and acinar cell death^30,31^. However, in the current experimental setting where we used human acinar tissue, it would be difficult to prove if trypsin activation was directly or indirectly related to our observations on cytokine release and second hit to the acinar cells. Nevertheless, the above observations from clinical studies explain, at least partly, why modalities found to be effective in experimental AP does not work optimally in patients. This, along with our results, also suggests that for patients with AP, definitive treatment modalities that target the early cytokine response rather than the initial acinar injury needs to be evaluated.

In conclusion, our study shows that in human biliary AP, pancreatic injury is associated with cytokine secretion by the injured acini, which in turn causes activation of circulating PBMCs. The activated PBMCs causes a second hit to the pancreas and further trigger a systemic inflammatory response, which likely determines the severity of the disease. In this manuscript, we have reported gross early mechanistic events in human AP. Our results highlight events that have potential therapeutic implications, and thus need to be explored in greater detail so that definitive treatment modalities could be developed.

## Material and Methods

### Study site and approval

The studies were conducted at the Asian Institute of Gastroenterology, which is a high volume referral center for pancreatic diseases. The study protocols were approved by the Asian Healthcare Foundation/Asian Institute of Gastroenterology Institutional Review Board (Reference no. AIG/AHF IRB 12/2011; dated 09/07/2011). Informed consent was obtained from all participants from whom pancreatic and blood samples were procured. All methods were performed in accordance with the relevant guidelines and regulations.

### Materials

Atropine, Carbachol, FAEE, Taurolithocholic acid disodium salt, Bovine serum albumin, and Collagenase IV from Clostridium histolyticum were obtained from Sigma-Aldrich (St. Louis, USA); Lipopolysaccharide from Escherichia was purchased from Sigma-Aldrich (Rehovot, Israel). The substrates BOC-Gln-Ala-Arg-7AMC and Z-Arg-Arg-7AMC hydrochloride were obtained from Sigma-Aldrich (Buchs, Switzerland), LDH cytotoxicity assay kit was obtained from G-Biosciences (St. Louis, USA). Hisep LSM 1077, Dulbeccos modified eagle medium, Penstrep, Phosphate buffered saline, Fetal bovine serum were purchased from Himedia laboratories (Mumbai, India). Cytometric bead array (CBA) human inflammatory cytokines kit was purchased from BD Biosciences, USA. Autozyme, an amylase quantification kit was purchased from Accurex Biomedicals (Thane, India); recombinant TNF-α was purchased from Peprotech (Rocky Hill, NJ USA). Polyclonal primary antibodies (anti IL-6, anti-TNF-α, anti-LC3, anti caspase-3) were purchased from Abcam (Cambridge, UK), while anti-amylase antibody was purchased from Santacruz (California, USA). All other chemicals were obtained either from Sigma-Aldrich (Germany, USA) or SDFCL Fine chemicals limited (Mumbai, India).

### Sample procurement and processing

Normal human pancreatic tissues were procured by the method described earlier^4^, from the operating rooms of Asian Institute of Gastroenterology Hospital, Hyderabad from patients who underwent Whipple’s surgery or distal pancreatectomy for the indications other than chronic pancreatitis and pancreatic malignancy. Briefly, after resection, a 1–3 cm^3^ sized pancreatic tissue was cut with a fresh scalpel from the transection margin of the intact pancreas. The cut piece of the pancreas was immediately washed thrice with oxygenated ice-cold HEPES buffer (containing 127 mmol/L NaCl, 4.7 mmol/L KCl, 1.0 mmol/L Na2HPO4, 10 mmol/L HEPES, 1.06 mmol/L MgCl2, 1.28 mmol/L CaCl2,10 mmol/L D-glucose) and then transferred to the laboratory in fresh oxygenated ice-cold HEPES containing trypsin inhibitor and sodium pyruvate. Once in the laboratory, extracellular fat was removed from the tissue and it was strictly ensured that the pancreatic tissue was processed for experiments within 10 mins of procurement.

Some of the tissues were minced into fragments of <0.5 mm^2^ without using collagenase, as described earlier^31^. Pancreatic fragments prepared by this method were reported to remain viable and functional for up to 24 hrs^32^. Remaining tissues were used for isolation of acinar clusters.

### Pancreatic acinar cluster isolation

Human pancreatic acinar clusters were isolated, as described earlier, after modifying the method for rodent acinar cell isolation^4^. Briefly, pancreatic tissue sample was injected at multiple sites with collagenase (200 U/mL). Following this the tissue was minced into small bits and transferred into fresh collagenase solution. The minced pancreata was then incubated in oxygenated HEPES medium containing collagenase solution at 37 °C with shaking for 30 minutes and pH adjusted to 7.4. After 10 minutes of incubation the collagenase was drained, and minced pieces were suspended in fresh HEPES. The suspension was then triturated and centrifuged at 1000 RPM for 1 minute. This step was performed thrice following which the suspension was filtered through a 140 mm nylon mesh. The filtered cells were resuspended in fresh HEPES and purified by sedimentation through 4% bovine serum albumin. The purified acinar clusters were washed thrice and finally dispersed in fresh HEPES or DMEM, which was used for experiments. All experiments were conducted at room temperature. As demonstrated earlier, pancreatic acini prepared by this method could be maintained in culture for 24–48 hrs^33^.

### Induction of pancreatic injury

Pancreatic fragments or acinar clusters were exposed to 500 μM TLCS, 50 μM FAEE or 50 μM caerulein for 1–2 hrs in oxygenated medium, after which they were subjected to histology and biochemical experiments. For histology studies, blocks were prepared with the exposed pancreatic tissue fragments or acinar clusters, which were then sliced into thin sections of 140 μm for H&E staining, IHC and IF. Media of the treated acinar fragments were collected and used for flowcytometry to assess the cytokines secreted by injured pancreatic tissue. The collected (conditioned) media was also used to activate PBMCs. For biochemical assays, acinar fragments were homogenized while acinar clusters were sonicated; and the resulting supernatant was used for the assays.

In order to evaluate the functional integrity of the acini, they were evaluated for a normal secretory response. Briefly, freshly prepared pancreatic acinar fragments were treated with incremental doses (1 μM to 1 mM) of carbachol for 15 and 30 minutes. In few experiments, the acinar tissues were pre-treated with 10 μM atropine for 20 mins after which the media was replaced with fresh HEPES and treated with carbachol. Once the incubation was over, amylase was estimated in the media and expressed as percent of total^5,10^.

### Measurement of pancreatic enzyme activity

Pancreatic amylase activity was measured in the medium of treated pancreatic tissue at 37 °C by using the auto analyser (Model: ERBA Chem-5 plus v2). Results were expressed as IU/ml after normalization.

Trypsin activity was measured at 37 °C by using BOC-Gln-Ala-Arg-MCA as substrate as described by Kawabata, *et al*.^34^. Fluorescence was measured with emission at 460 nm after excitation at 350 nm on the Fluroskan Assent (Thermo Scientific, USA). Results were analyzed and expressed as total trypsin activity per mg of protein.

Cathepsin B activity was measured by using Z-Arg-Arg-7AMC as substrate according to method described by Mcdonald & Ellis^35^. Hydrolysis of substrate by cathepsin-B to form B-naphthalamide was measured spectroflurometrically (Ex: 350 nm and Em: 460 nm). Final results were expressed as total cathepsin B activity per mg of protein.

### Identification and quantification of acinar cell injury

Pancreatic tissue injury was identified and quantified by Hemotoxylin & Eosin staining of formalin fixed paraffin embedded (FFPE) tissue sections. Swollen and lightly stained cytoplasm, nuclear pyknosis, loss of membrane integrity were considered as histological markers of tissue injury. The histological sections were examined under light microscope (Olympus CX 41) and percentage of injury was expressed by calculating the injured area in at least 5 randomly selected images per histology sections (n = 3) from each experiment in the ImageJ software. All imaging studies (including IHC and IF) were conducted by a single experienced senior research pathologist who was blinded to the experimental grouping.

### Immunohistochemistry

Immunoperoxidase staining was performed on the FFPE tissues and cell-blocks. Sections were deparaffinized through different concentrations of xylene and ethanol followed by sodium citrate antigen-retrieval. Peroxidase blocking was performed by 3% H_2_O_2_. Tissue sections were incubated with Rabbit polyclonal LC3 primary antibody at 4 °C overnight in 1:1000 dilution, followed by goat polyclonal anti-rabbit secondary antibody (1:2000 dilution). Antibody staining was developed using the DAB detection system, and accompanied by hematoxylin counterstain and was examined under light microscope (Olympus CX 41). We quantified positive cells in the cell blocks by taking at least 5 images from every experiment that were randomly picked and counted the total number of positive cells per 100 nuclei, and expressed the numbers as mean (SEM).

### Immunofluorescence

IF was conducted using thin sections of human pancreatic fragments and acinar cell-blocks. Briefly, the sections were deparaffinised with xylene following which they were washed and fixed in 100% methanol and incubated with primary antibody (1:1000) at 4 °C overnight, followed by fluoroscent tagged secondary antibody (1:2000) in dark at room temperature. After several steps of washing, final incubation with DAPI for 15 min was performed. At least 5 randomly selected images per histology sections from each experiment (n = 3) were viewed in the Olympus IX71 fluorescence microscope, and fluorescent images captured using the CARVII bioimager (BD Biosciences) mounted with the IPLAB software (BD Biosciences). We quantified IL-6 or TNF-α positive cells per 100 amylase positive cells and expressed as mean (SEM).

### Transmission electron microscopy

TEM was performed using less than 0.5 mm^3^ bits of TLCS treated pancreatic fragments as described earlier^36^. After completion of treatment the pancreatic fragments were immersed in modified Karnovsky’s solution [2.5% glutaraldehyde, 2% paraformaldehyde in 0.1 M phosphate buffer, pH 7.2]. After 48 hrs of Karnovsky fixation tissues were transferred to PBS and transported for transmission electron microscopy. The tissues were post-fixed in 1% OsO_4_, dehydrated in ascending grades of acetone, embedded and blocked in araldite CY212. After determining the regions of interest on toluidine blue stained sections, 50–60 nm sections were cut on a Reichert-Jung [Leica, Massachussetts, USA] ultracut microtome and collected on 300 mesh copper grids. The sections were stained with uranyl acetate and lead citrate and viewed under Philips Morgagni 268 D TEM [Field Emission Inc., Netherlands]. A MegaView III CCD camera that was integrated with the iTEM software [Olympus Soft Imaging Solutions, Münster, Germany] acquired the photographs, with instrument-calibrated scale bars.

### Lymphocytes isolation and *in vitro* stimulation

Peripheral blood mononuclear cells (PBMCs) were prepared using Hisep (Himedia, Mumbai, India) and cells were adjusted to 1 × 10^6^ cells per ml in DMEM (Sigma Aldrich, St. Louis, USA) with 10% fetal bovine serum, 1% penstrep, 2 mM glutamine. PBMCs were plated in 12 well plates and stimulated with conditioned culture media of injured acinar cells from the same patient for 18 hrs. Unstimulated cells were taken as controls. After 18 hrs of stimulation, the acinar induction media was replaced by fresh media and assessed for detection and quantification of cytokines released by activated PBMCs after another 6 hrs.

We also conducted experiments wherein pancreatic acinar tissues were treated with conditioned medium in which PBMCs were activated by LPS (1 μg/ml). The conditioned medium was replaced by fresh media after 12 hrs of activation, and cytokines liberated by the pancreatic tissue were measured in the fresh media at the end of 18 hrs. Freshly prepared acini were also treated with 100 ng/ml of recombinant TNF-α for 3 hrs and the medium was assessed for IL-6.

### Quantification of cytokines

Cytokines in experimental media and patient’s plasma were quantified in BD FACS ARIA II system. BD cytometric bead array (CBA) human inflammatory cytokines kit (BD Biosciences, USA) was used to quantify the cytokines levels in the samples according to manufacturer’s instructions.

### Patients and controls

In order to elucidate the early cytokine dynamics in patients with AP, we prospectively enrolled 45 consecutive patients with acute biliary pancreatitis who were admitted within 72 hrs of disease onset. We also recruited 10 blood donors [mean age (SD) 37.6 (4.1); all males] as controls for plasma cytokine assay. We recorded the following parameters: demographic characteristics (age, gender), disease severity (defined according to the Revised Atlanta Classification), presence of SIRS, details of organ failure, interventions required, and in-hospital deaths. We defined early severe AP (ESAP) as development of OF within 7 days of onset and fulminant AP as development of OF within 72 hrs of onset, as described earlier^18,19^.

### Statistical analysis

A database was generated in Microsoft Excel for Mac (Ver. 14.6.9, Redmond, USA) and all statistical analyses were performed using SPSS (Ver. 20; Chicago, USA). Continuous variables were expressed as mean with standard error of mean (SEM), while the categorical variables were represented as proportions (percentage). Distribution of continuous data was tested by the goodness-for-fit test, and non-normally distributed data were log transformed before analyses. For comparing continuous variables between two groups, the students ‘t’ test or Mann-Whitney U test was used as appropriate. Whenever there were three groups, comparison was done using either ANOVA or Kruskal Wallis test as appropriate. Comparison of categorical variables was done using the Fischer’s Exact or the chi square tests. A two-tailed ‘p’ value of <0.05 was considered statistically significant.

## Electronic supplementary material

Supplementary data
