# Supplementary material for: Acinar injury and early cytokine response in human acute biliary pancreatitis
Source: Sci Rep. 2017 Nov 10;7:15276. doi: 10.1038/s41598-017-15479-2 (PMC5681596; doi:10.1038/s41598-017-15479-2)

Supplementary materials

Acinar injury and early cytokine response in human acute biliary pancreatitis.

Aparna Jakkampudi, Ramaiah Jangala, Ratnakar Reddy, Sasikala Mitnala, G Venkat Rao, Rebala Pradeep, D Nageshwar Reddy, Rupjyoti Talukdar.

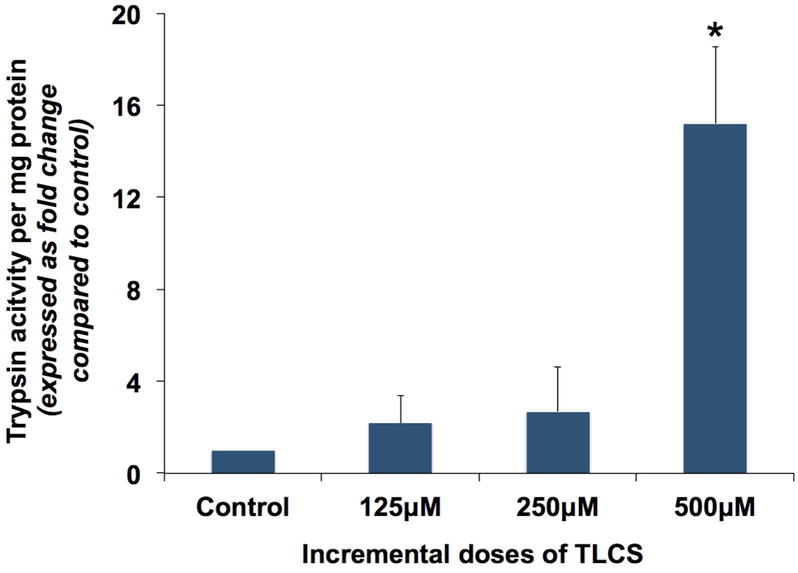

Supplementary Figure S1:

Effect of increasing doses of TLCS on trypsin activation in human pancreatic acini. Human pancreatic acinar clusters were exposed to different doses of TLCS for 1 hr followed by evaluation of trypsin activation.

Significantly high trypsin activity was observed at a dose of 500M of TLCS, while there was no increase in trypsin activity at doses of 125M and 250M.

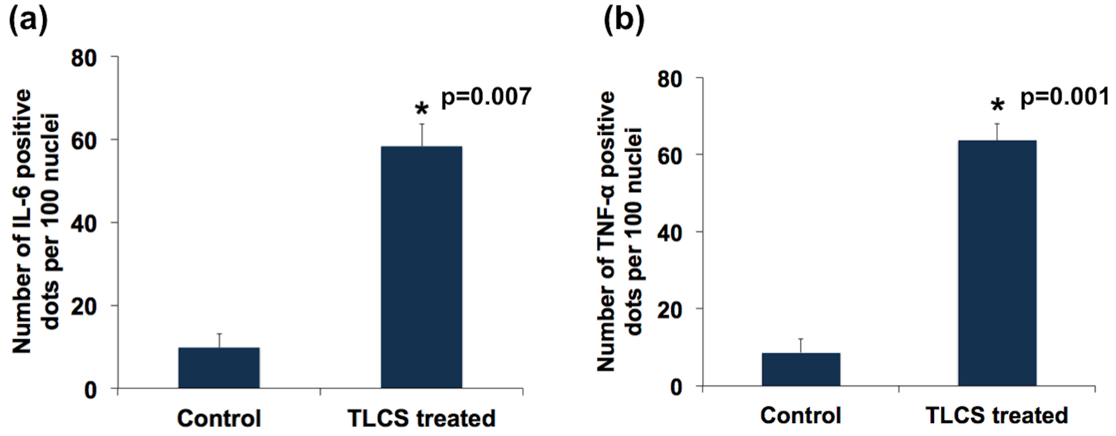

Supplementary figure S2:

Histograms showing the quantitative representation of IL-6 (a) and TNF-α (b) within human pancreatic acinar cells after treatment with 500μM TLCS for 6hrs. Error bars indicate SEM.

Supplementary figure S3:

Representative FACS images showing a panel of cytokines in patients with mild AP (MAP), moderately severe AP (MSAP) and severe AP (SAP).
The cytokines shown in the images are (from top to bottom):
IL-8, IL-1b, IL-6, IL-10, TNF-α, and IL-12p70.

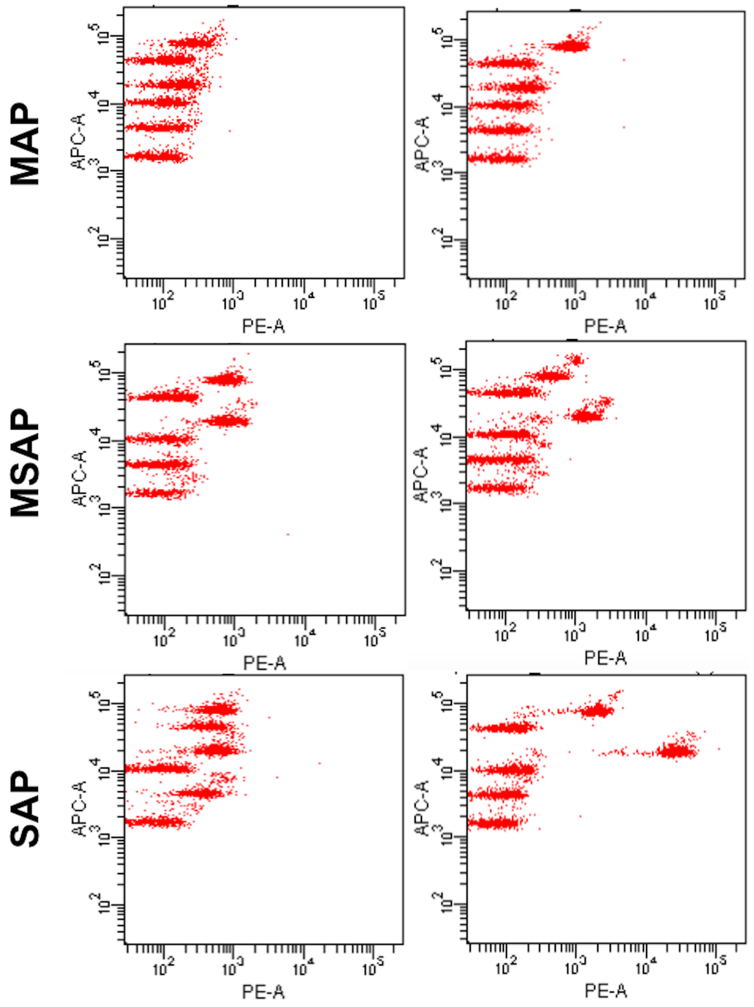

Supplement: Supplementary file 1 — Supplementary data [file 41598_2017_15479_MOESM1_ESM.pdf]
